# Efficacy of Aflibercept as initial treatment for neovascular age-related macular degeneration in an Iraqi patient sample

**DOI:** 10.25122/jml-2022-0356

**Published:** 2023-02

**Authors:** Zaid Rajab Hussein, Sufyan Khalid Omar, Rasha Abdulelah Mustafa Alkazraji, Ahmed Nezar Alsamarrai, Hayder Sabah Alrubaye, Hany Akeel Al-hussaniy

**Affiliations:** 1 Department of Ophthalmology, Ibn Al-Haithem Teaching Eye Hospital, Baghdad, Iraq; 2 Department of Pharmacology, College of Medicine, University of Baghdad, Baghdad, Iraq; 3 Dr. Hany Akeel Institute, Iraqi Medical Research Center, Baghdad, Iraq; 4 Department of Pharmacy, Bilad Alrafidain University College, Diyala, Iraq

**Keywords:** Aflibercept, vascular endothelial growth factor (VEGF), anti-VEGF, neovascular age-related macular degeneration (AMD), prospective study

## Abstract

Age-related macular degeneration (AMD) is a progressive degenerative eye disorder that primarily affects individuals over 50. It causes gradual loss of central vision and can lead to irreversible severe visual loss if left untreated. AMD is a leading cause of blindness in the developed world. This study aimed to investigate the effects of a loading dosage of intravitreal Aflibercept on functional and morphological responses in neovascular AMD, considering demographic characteristics and the link between AMD-related retinal symptoms at presentations. A prospective interventional study was conducted from November 2021 to September 2022 on a sample of Iraqi patients with neovascular AMD who had active choroidal neovascularization (CNV) lesions confirmed by OCT-A and received intravitreal Aflibercept 2mg injection as initial therapy (3 loading doses). Best-corrected visual acuity (BCVA) was used to measure functional responses, and central macular thickness (CMT) and maximum area of the retinal thickness (MART) (by SD-OCT) were used to measure morphological responses. The study included 48 patients (57 eyes) with active neovascular AMD. The mean difference of BCVA in log MAR (0.2 ± 0.7) significantly improved from 1.3±0.7 at baseline to 1.1±0.8 after loading Aflibercept (P=0.034). The mean difference in CMT 113.6 ± 125.9 was statistically significant (P<0.0001). Also, the mean change in MART significantly decreased from 444.2 ± 127.1 µm at baseline to 348.7±74.5 µm (p < 0.0001) after loading Aflibercept. This study demonstrated that Aflibercept is a functionally and anatomically successful treatment for neovascular AMD.

## INTRODUCTION

Age-related macular degeneration (AMD) is a degenerative disorder that is acquired, irreversible, and complex. AMD is described by the gradual degradation of retinal photoreceptors and retinal pigment epithelium (RPE) and the thickening of Bruch's membrane (BM). AMD also mainly affects the choriocapillaris in the retina's macula portion, leading to the loss of central vision [1, 2]. This impairs the patient's ability to read, drive, identify faces, and perform other necessary everyday duties, which can negatively influence their quality of life [3]. There are two types of AMD: dry or non-neovascular AMD and wet or neovascular AMD. The main characteristics of non-neovascular AMD are changes to the retinal pigment epithelium (RPE), small, spherical extracellular substance deposits of yellow or white color called drusen, and atrophic areas that merge to form geographic atrophy.

On the other hand, wet AMD is characterized by the proliferation of choroidal neovascular membranes or the development of irregular vessels in the retina and behind the RPE. Because only approximately 10% of AMD patients have the exudative type, serious visual impairment and blindness occur in up to 80%-90% of cases [4]. Wet age-related macular degeneration (AMD) is the most aggressive type of AMD and the leading cause of legal blindness [5,6]. Patients with wet AMD in one eye have a 12% chance of getting wet AMD in the other [7]. If left untreated, it can cause severely impaired vision and blindness [8]. The current gold standard for treating neovascular AMD is intravitreal anti-vascular endothelial growth factor (VEGF) therapy [9].

On November 18, 2011, the FDA approved Aflibercept (EYLEA®, Regeneron Pharmaceutical Inc, and Bayer). Initially known as VEGF trap-eye, it is an anti-VEGF drug for treating wet AMD [10]. The VIEW 1 and VIEW 2 studies showed that both medications, Aflibercept and Ranibizumab, were equally effective in visual acuity and anatomic outcomes [9]. Pharmacological tests of Ranibizumab and Aflibercept have demonstrated a greater affinity for VEGF-A binding [11] and a considerably longer ligand binding activity [12]. Structurally, Aflibercept is a 115 kDa soluble decoy receptor produced by VEGFR-1 (second binding domain) and VEGFR-2 (third binding domain) attached to the Fc portion of human immunoglobulin (IgG), giving it a significantly greater affinity than the natural receptors [1,4]. Aflibercept binds to all VEGF isoforms, placental growth factors (PIGF 1 and 2), and VEGF-B, with a particularly high affinity for VEGF-A165, 94 times larger than Ranibizumab and 120 times greater than Bevacizumab [13].

Aflibercept serves as an anti-VEGF therapy through its mechanism as a soluble decoy receptor, capable of binding to all isoforms of VEGF-A and placental growth factors. The activation of the tyrosine kinase receptors, VEGFR-1 and VEGFR-2, located on the surface of endothelial cells by VEGF-A results in the induction of neovascularization and increased vascular permeability. Aflibercept counters these effects by forming a drug-ligand complex with VEGF, effectively blocking the interaction between the ligand and receptor and suppressing angiogenesis [14].

Optical coherence tomography (OCT) has been widely used to examine the posterior segment of the eye in patients with neovascular age-related macular degeneration (AMD), and the assessment of lesion structure using OCT has become an important step in the clinical decision-making process [15]. The therapy and frequency of treatment for wet AMD patients depend on signs of disease activity based on OCT, such as intraretinal and subretinal as well as subretinal pigment epithelium (RPE) fluid. Recent advancements in OCT technology have resulted in enhanced speed and resolution, allowing the identification of small morphological changes to the retinal layers [16].

## MATERIAL AND METHODS

This prospective study, conducted at the Ibn Al-Haitham Teaching Eye Hospital in Baghdad, Iraq, evaluated the effectiveness of Aflibercept 2mg/0.05ml administered monthly for three months in treating wet age-related macular degeneration (AMD) between November 2021 and September 2022.

The criteria for inclusion in this study were:

- Patients with wet age-related macular degeneration (AMD) diagnosis, confirmed by clinical examination, optical coherence tomography (OCT), and optical coherence tomography angiography (OCTA);
- Patients aged 50 years or older;
- Willingness to receive three intravitreal injections of Aflibercept.

Exclusion criteria included:

- Previous treatment for wet AMD with Photodynamic therapy (PDT) or other intravitreal injections;
- Advanced AMD-like disciform scar or choroidal neovascularization caused by degenerative myopia or Angioid streaks;
- Retinal angiomatous proliferation or polypoidal choroidal vasculopathy;
- Other conditions that may affect the quality of the OCT, such as cataracts and vitreous hemorrhage;
- Complications after injection, such as endophthalmitis, retinal detachment, or vitreous hemorrhage that may affect vision.

Newly diagnosed neovascular AMD patients with no previous treatment of AMD were included in this study. All patients had pre-injection data recorded, including demographic data and clinical data such as best corrected visual acuity (BCVA) and central macular thickness (CMT), and the maximum area of a retinal thickness (MART). Following the diagnosis of wet AMD with active choroidal neovascularization (CNV) lesions confirmed by optical coherence tomography angiography (OCT-A), patients were given three loading doses of Aflibercept. The response to treatment was evaluated both functionally and anatomically using standard optical coherence tomography (SD-OCT). Patients were re-examined one month after the third injection to assess the response to Aflibercept treatment and the relations of different factors (presence or absence of intraretinal fluid, subretinal fluid, intraretinal hemorrhage, subretinal hemorrhage, and retinal pigmented detachment) to the response of aflibercept treatment.

Data analysis was conducted using the Statistical Package for the Social Sciences (SPSS, version 25) and Microsoft Excel 2019. Descriptive analysis included the calculation of frequencies and percentages for categorical variables and mean values with standard deviations for continuous variables. The chi-square test was used to compare proportions between groups. The changes in the mean values of BCVA, CMT, and MART from baseline to post-loading were compared using a paired t-test. The significance level was set at P≤0.05.

## RESULTS

In the current study, 57 eyes from 48 patients were included. Their ages ranged from 50 to 85 years, with a mean age of 68.3±8.6. Twenty-eight were males (58.3%) with a male-to-female ratio of 1.4:1. Although the mean age of males was slightly higher than females (69.2±8.4 vs. 67.4±8.9, respectively), the difference was not statistically significant (Student’s t-test, df=47, P=0.5) (Table 1).

**Table 1:** Mean age (in years) of patients.

Gender	Age (in years)		P-value
	Range	Mean±SD	
**Male**	50-82	69.2±8.4	0.5*
**Female**	53-85	67.4±8.9	
**Total**	50-85	68.3±8.6	

* The differences between mean ages were not significant; Student’s t-test, df=47, P=0.5

### Ocular characteristics of the treated eye

**Figure 1:**
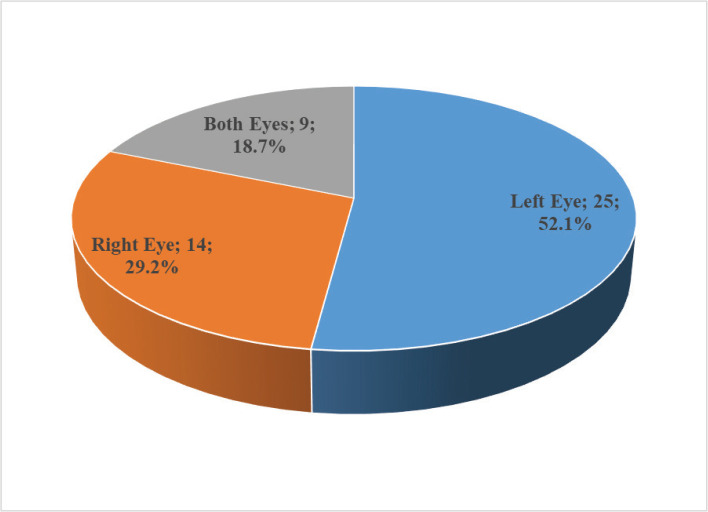
Distribution of patients by affected eye.

Among the 48 patients included in the current study, the left eye was affected in 25 (52.1%) patients, the right eye in 14 (29.2%) patients, and both eyes (right and left) in 9 (18.7%) patients (Figure 1).

### Comorbidities

30 (62.5%) patients had hypertension, of whom 16 (53.3%) were female. Only 10 (20.8%) had diabetes, with an equal distribution of males and females. Furthermore, 16 (33.3%) were current smokers, all male (Table 2).

**Table 2:** Patient characteristics.

Characteristics	No.	%
**Age in years**		
≤70	24	50
>70	24	50
**Gender**		
Males	28	58.3
Females	20	41.7
Chronic Diseases*		
Hypertension	30	62.5
Diabetes Mellitus	10	20.8
**Smoking status**		
Smoker	16	33.3
Not smoker	32	66.7

* Some patients had both hypertension and diabetes.

### Evolution of visual acuity

**Figure 2:**
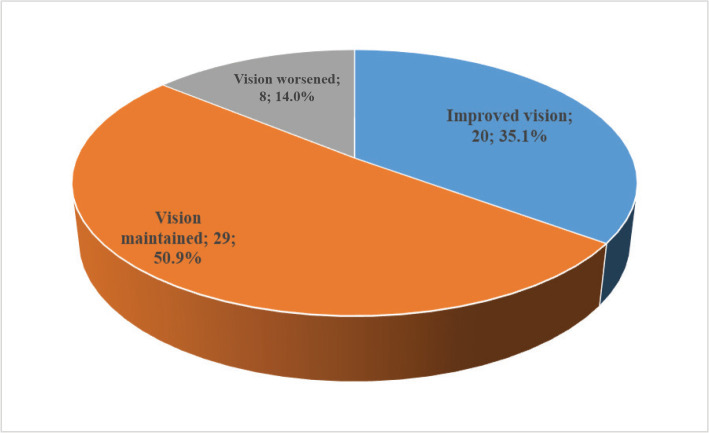
Patient's response to treatment according to BCVA.

Best-corrected visual acuity (BCVA) was measured before and after treatment. As shown in Figure 2, an improved vision was observed in 20 (35.1%) eyes, remained unchanged in 29 (50.9%) eyes, and worsened in 8 (14.0%) eyes. The mean difference in BCVA was statistically significant (Paired t-test, P=0.034) (Table 3).

**Table 3:** Mean BCVA before and after treatment and mean differences.

BCVA	Range	Mean±SD	P-value
**Before**	0.2-2.3	1.3±0.7	0.034*
**After**	0-2.3	1.1±0.8	
**Mean difference**	-1.5-1.8	0.2±0.7	

* The mean difference in BCVA before and after treatment was statistically significant; Paired t-test, df=56, P=0.034.

### Evolution of central macular thickness (CMT) and maximum area of the retinal thickness (MART)

The results of the CMT measurement showed that a good response was observed in 37 (64.9%) eyes, while 15 (26.3%) eyes maintained their response, and only 5 (8.8%) eyes had a poor response. The data from the pre-and post-treatment CMT measurements revealed a decrease from 394.2 to 281.2, with a mean difference of 113.6 ± 125.9, which was statistically significant (Paired t-test, P<0.0001) (Table 4). Similarly, when measuring mean valu of MART, it was found that a good response was present in 39 (68.4%) eyes, maintained in 17 (29.8%) eyes, and only one (1.8%) eye showed a poor response. The mean MART decreased from 444.2 before treatment to 348.7 after treatment, with a mean difference of 95.4 ± 97.1, which was also found to be statistically significant (Paired t-test, P<0.0001) (Table 4).

**Table 4:** Mean CMT and MART before and after treatment.

Measurement	Range	Mean±SD	P-value
**CMT**			
Before	200-813	395.2±131.2	<0.0001*
After	167-485	281.2±70.9	
Mean difference	-144-526	113.6±125.9	
**MART**			
Before	214-811	444.2±127.1	<0.0001*
After	243-711	348.7±74.5	
Mean difference	-79-436	95.4±97.1	

* The mean difference before and after treatment was statistically significant; Paired t test, df=56, P<0.0001.

Regarding retinal signs of CNV at initial presentation, Table 5 shows that the most prevalent was subretinal fluid (SRF), observed in 41 (71.9%) eyes. This was followed by intraretinal fluid (IRF), which was present in 30 (52.6%) eyes, and the least common was subretinal hemorrhage (SRH), identified in only 2 (3.5%) eyes.

**Table 5:** Retinal signs at presentation with choroidal neovascularization (CNV) lesions.

Variable	No.	%*
**SRF**	41	71.9
**SRH**	2	3.5
**IRF**	30	52.6
**IRH**	3	5.3
**PED**	11	19.3

* Some eyes have more than one complication.

### Variables associated with visual result

We analyzed the relationship between treatment outcome and active choroidal neovascularization (CNV) lesions at the start of treatment. There were statistically significant associations between the type of response to treatment and the presence of SRH and IRF (X^2^ test, df=1, P≤0.05) (Table 6).

**Table 6:** Association between BCVA and active choroidal neovascularization (CNV) lesions.

Factors	BCVA				P-value
	Improved (20)		Maintained and Worsened (37)		
	No.	%	No.	%	
**SRF**					
Yes	15	36.6	26	63.4	0.7
No	5	31.3	11	68.7	
**SRH**					
Yes	2	100.0	0	0.0	0.05*
No	18	32.7	37	67.3	
**IRF**					
Yes	7	23.3	23	76.7	0.04*
No	13	48.2	14	51.8	
**IRH**					
Yes	1	33.3	2	66.7	0.9
No	19	35.2	35	64.8	
**PED**					
Yes	5	45.5	6	54.5	0.4
No	15	32.6	31	67.4	

* The association was statistically significant; X^2^ test, df=1, P≤0.05.

### Variables associated with the reduction of CMT

An analysis of the relationship between the treatment response and the initial presence of CNV lesions, as determined by a decrease in CMT, revealed a statistically significant association with the presence of IRF at presentation (X^2^ test, df=1, P≤0.05) (Table 7).

**Table 7:** Association between CMT and retinal signs with CNV lesions.

Factors	CMT				P-value
	Improved (37)		Maintained and Worsened (20)		
	No.	%	No.	%	
**SRF**					
Yes	25	61.0	16	39.0	0.3
No	12	75.0	4	25.0	
**SRH**					
Yes	1	50.0	1	50.0	0.6
No	36	65.4	19	34.6	
**IRF**					
Yes	23	76.7	7	23.3	0.04*
No	14	51.9	13	48.1	
**IRH**					
Yes	2	66.7	1	33.3	0.9
No	35	64.8	19	35.2	
**PED**					
Yes	5	45.4	6	54.6	0.1
No	32	69.6	14	30.4	

* The association was statistically significant; X^2^ test, df=1, P≤0.05.

## DISCUSSION

Intravitreal anti-VEGF therapy is currently the drug of choice for neovascular AMD. This study found that using intravitreal Aflibercept with a loading dose improves functional and structural outcomes in newly diagnosed patients with wet AMD. The average age of the study population was 68.3 ± 8.6 years, ranging from 50 to 85 years. This is lower than the average age seen in other clinical trials [17, 18]. A review published in 2018 also noted that the average age in clinical trials is typically higher, at 76-77 years [19]. This may be because the rate of aging in the European Union is high, with 26.7% of the population over the age of 65, according to data from the National Statistics Institute in 2014.

The current study found that after a 3-month loading period of intravitreal Aflibercept injections, there was a statistically significant improvement in BCVA with a mean difference of 0.2±0.7, improving from 1.3±0.7 at baseline to 1.1± 0.8 at 4 months (P = 0.034). This improvement was observed in 20 (35.1%) eyes, with no change in 29 (50.1%) eyes and a deterioration in 8 (14%) eyes. These findings are consistent with previous studies [20,21]. These results suggest an 'initiation phase' in which there is a fast gain in maintaining visual acuity in response to the first three treatments, followed by a phase of stability in which the maintained visual acuity remains stable.

According to the MARINA and ANCHOR studies, BCVA improved significantly in individuals with neovascular AMD following the first 3 consecutive monthly injections of Ranibizumab [20,-22]. We observed a similar response in the SEVEN-UP study, which is an extension of the MARINA, ANCHOR, and HORIZON studies, with an initial gain in the monthly guideline of +11.2 ETDRS letters [18]. Moreover, our findings agree with the CATT study [17], where they observed a visual gain of +9 to +10 letters of ETDRS in the first two years of therapy. In clinical trials, intravitreal Aflibercept, after three initial monthly doses, produced similar efficacy outcomes as Ranibizumab [9,13]. Data from the CLEAR-IT 2 study showed that significant anatomic and visual improvements were achieved during the 12-week fixed dosing phase, and pro re nata (PRN) dosing was maintained [23,24].

In the current study, we observed an improvement in visual acuity (> +0.2 LogMAR) in 35.1% (20 eyes) of patients, which is slightly higher than the results from the CATT trial, which showed that 28-31% of patients had improved visual acuity at the start of follow-up, but this percentage decreased to 17.6% after 5 years [25,17]. Similar results were also observed in the Tufail *et al*. study, where 30% of patients had improved visual acuity in the first and second years and 29% in the third year [26]. We noticed a similar response to the VIEW research [13], which observed a visual gain of 31.6%, 31.2%, and 33.4% after 96 weeks of follow-up. In general, our visual improvement results are comparable to clinical trials and studies of routine clinical practice with a similar treatment regimen, regardless of the medication used, using the follow-up time factor and a fixed guideline.

Our study found that 14% (8 eyes out of 57) of cases experienced a decrease in visual acuity (< -0.2 LogMAR), which is lower than the 33.8% reported in the SEVEN-UP study compared to the monthly guidelines (MARINA, ANCHOR) [21,18]. On the other hand, our results were much higher than the CATT findings at 2 years (6.7%) [25]. Moreover, the results of the meta-analysis by Chong *et al*. [27] are also lower than our study, where the percentage of cases with visual loss ranged from 2.2% to 9.8%, despite the included studies having follow-up times of 1 to 2 years, compared to our study's 4-month duration. Based on these results, we think that the follow-up time factor and baseline visual acuity are key, and numerous samples were also affected.

**Figure 3:**
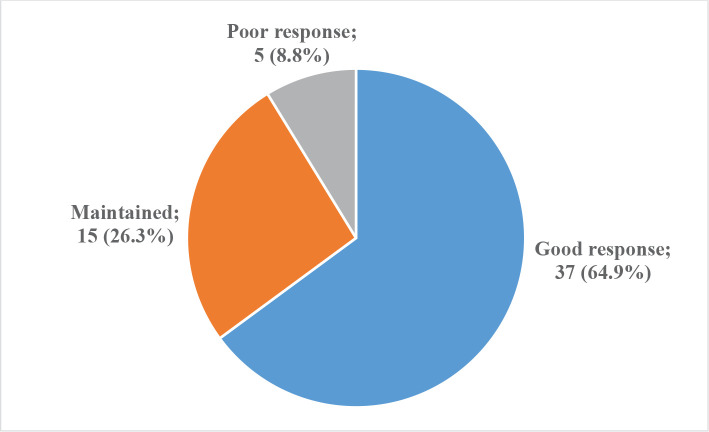
Patient's response to treatment according to CMT.

In this study, we demonstrated that central macular thickness decreased across the macula, not just in the foveal center, in eyes with neovascular AMD treated with Aflibercept (Figure 3). With three monthly intravitreal Aflibercept injections, the mean CMT significantly decreased from 395.2 ± 131.2 µm at baseline to 281.2 ± 70.9 µm (P-value < .0001) at four months. Also, the mean change in the MART in all 57 eyes treated significantly decreased from 444.2 ± 127.1 µm at baseline to 348.7 ± 74.5 µm (p< 0.0001) at 16 weeks. This agrees with VIEW 1 and VIEW 2 trials which also demonstrated reduced CMT and better BCVA following three first monthly doses of Aflibercept and Ranibizumab. In the first year, the decrease in CMT ranged from - 123 m to -139 m, with the greatest reduction observed with Aflibercept 2 mg administered monthly and biweekly. At 96 weeks, the reduction ranged from -113 m to -133 m compared to the baseline CMT. The data indicates that the largest reduction in CMT occurs within the first year of treatment [13].

Moreover, the CATT study results show an apparent decrease in total macular thickness and significant changes compared to the baseline and final CMT at one year [17,25]. Our study found that the greatest decrease in macular thickness occurred within the first three months, consistent with other publications.

In our study, patients with IRF had significantly worse visual acuity (30 eyes) than patients without IRF (27 eyes) (P-value=0.04). Similarly, the presence of IRF has been linked to poorer vision both at the beginning and during therapy in major clinical studies such as CATT [28], VIEW [29], and EXCITE research [30]. Moreover, recent investigations assessed IRF volume and found a similar association [31,32]. In contrast to SRF, numerous studies have demonstrated that IRF has a strong negative impact on BCVA [33,34]. According to Ritter *et al*., IRF has the worst outcome for functional improvement, especially in eyes with the lowest initial visual acuity (VA) [35]. Previous studies indicated that patients with IRF at baseline must aggressively manage fluid persistence using stringent treatment regimens to prevent blindness caused by photoreceptor loss [36]. IRF causes alterations in bipolar axons, leading to neurosensory impairment [37]. The VIEW study found that at 1 year, IRF and diffuse epiretinal proliferation (DEP) were associated with reduced visual gain (-2.77 and -1.88 letters) compared to SRF, which was associated with a gain of +2.11 letters. Therefore, active lesions with IRF have worse visual outcomes than those with little hemorrhage, pigment epithelial detachment (PED), or SRF [13].

In this study, we evaluated the influence of SRF on visual and anatomic results in eyes with wet AMD. The presence of SRF was not related to decreased or increased visual acuity compared to the absence of SRF, which was not statistically significantly different from the absence of SRF (p = 0.7). Similarly, the EXCITE [30] and VIEW studies [13] also did not observe this relationship. The CATT study found that patients with better initial visual acuity had better visual outcomes and were able to preserve them throughout the first, second, and fifth years [29,38]. The accumulation of hyperreflective fluid above the RPE layer is known as subretinal fluid. According to several studies, the presence of SRF at baseline and after a year of treatment did not significantly increase visual acuity [39-41]. The presence of residual SRF may not always indicate continued neovascular activity. Instead, it could be RPE dysfunction that causes SRF buildup, similar to central serous chorioretinopathy [42]. This could suggest that the CNV lesion in eyes with subfoveal SRF is extrafoveal located and has less influence on BCVA. Further studies are needed to determine the pathophysiologic foundation of the positive events linked with SRF and to confirm these results.

According to our study, pigment epithelium detachment (PED) was not statistically linked to a lower visual acuity (P=0.4). Our findings agree with others [43-45]. PED's response to therapy has not been associated with visual outcomes. In contrast, the presence of PED has been linked to reduced visual acuity and worse baseline vision in wet AMD, according to a study by Ying *et al*. [46]. Furthermore, patients with a PED at baseline who acquired IRF during follow-up had the worst visual acuity of any anatomic parameter combination, according to a post-hoc analysis of the VIEW research [28]. Our study found that only 19.3% of the 11 eyes had PED, a much lower percentage than in other studies. For example, the EXCITE research reported that 74% to 85% of eyes had PED [34] and 75.8% in the VIEW trial [13]. Additionally, the height of the PED increases the risk of RPE rupture, which can worsen visual outcomes.

In this investigation, the association between the visual acuity of patients with subretinal hemorrhage (SRH) and those without SRH was significantly worse (P=0.05). However, there were only two patients with SRH at the onset due to blood buildup between the neurosensory retina and the retinal pigment epithelium (RPE) from the choroidal inside the macular area in neovascular AMD. Our findings are in agreement with Daniel *et al*., in a posthoc analysis [47], where increased hemorrhage (>1 disk diameter) was shown to be a risk factor for scar development, suggesting that the poor visual prognosis of these massive hemorrhagic lesions may be related to the risk of scar formation.

The study found a statistically significant association between a decrease in central macular thickness and a decrease in IRF. The persistence of IRF in 23.3% of eyes is considered a key factor in the progression of central fibrosis and deterioration of visual acuity. Therefore, it is important to be aggressive and intolerant of the persistence of fluid by applying strict treatment regimes. This finding agrees with Ho *et al*.'s study, which found a decrease of 50% in IRF at 6 months (from 26 to 13 eyes) [48]. In addition, a systematic review found that a reduction in SRF at 12 weeks is a strong predictor of good visual outcomes in wet AMD. However, patients with PED and IRF achieved smaller visual gains, and their treatment intervals should be cautiously extended [49]. Overall, it is clear that the presence of intraretinal fluid in wet AMD leads to worse visual outcomes.

In our study, the presence of subretinal fluid on OCT was not correlated with an increase or decrease in central macular thickness (p=0.3), which agrees with another research [50]. This research demonstrates that the presence of SRF did not affect visual and anatomic outcomes following anti-VEGF therapy. Also agreeing with a posthoc analysis, mean CMT with SRF decreased by -81.1 µm and -89.2 µm without fluid and with fluid, respectively [51]. According to Golbaz *et al*., SRF was more frequently linked to recurrent disease [52]. Despite confounding characteristics, SRF frequently suggests lesion activity and can be quickly noticed with OCT. However, if SRF does not diminish after intravitreal injections of anti-VEGF, it should be thought of as a degenerative phenomenon or the result of chronic RPE decompensation [53]. The pathomechanism leading to SRF's beneficial effects has not yet been clarified.

Our study showed no anatomically observed decline in PED height (P-value = 0.1). These findings corresponded with those of Ach *et al*. [54], who assessed 28 eyes treated for PED with bevacizumab and found stable visual acuity and no significant decline in PED. Additionally, the VIEW study shows that PED resolves poorly and ineffectively less than other morphologic variables, even when the strongest anti-VEGF drugs are used [55]. Additionally, despite the decreases in retinal fluid, PED height appeared to be stable or to diminish gradually in the CATT research [33]. Age-related abnormalities trigger the suggested PED in the Bruch membrane, and Bruch membrane thickness increases with age due to fat and abnormal material buildup. This accumulation lowers the hydraulic conductivity of the Bruch membrane-choroid complex, resulting in a diminished ability for fluid exchange between the choroidal and RPE compartments [56]. In contrast, Chen *et al*. [57] discovered stable or increased visual acuity in PED patients who received intravitreal bevacizumab over a 6-month follow-up period, despite the PED's continuous presence. The contrast between functional and morphological outcomes in PED could be linked to photoreceptor function loss due to long-term RPE separation [58].

IRH and SRH were observed in very few patients (3 (5.3%) eyes and 2 (3.5%) eyes, respectively), and the association with reduced central macular thickness was not significant.

## CONCLUSION

This study showed that Aflibercept was effective in treating patients with wet AMD both functionally and anatomically after loading dosages. The presence of intraretinal fluid at presentation harmed the visual response to treatment, whereas all other factors had a negligible impact on how well patients with wet age-related macular degeneration responded to a loading dose of Aflibercept. As a result, intraretinal fluid leads to worse visual outcomes. Finally, our research found that Aflibercept loading dosages improve visual acuity and reduce central thickness. The development of central fibrosis and the decline in visual acuity can be largely attributed to the persistence of IRF in 23.3% of the eyes. It is essential to follow tight treatment regimens and to be aggressive and intolerant of the persistence of fluid for this reason.
